# Urinary metabolomics reveals glycemic and coffee associated signatures of thyroid function in two population-based cohorts

**DOI:** 10.1371/journal.pone.0173078

**Published:** 2017-03-02

**Authors:** Nele Friedrich, Maik Pietzner, Claire Cannet, Betina H. Thuesen, Torben Hansen, Henri Wallaschofski, Niels Grarup, Tea Skaaby, Kathrin Budde, Oluf Pedersen, Matthias Nauck, Allan Linneberg

**Affiliations:** 1 Research Centre for Prevention and Health, The Capital Region of Denmark, Glostrup, Denmark; 2 Institute of Clinical Chemistry and Laboratory Medicine, University Medicine Greifswald, Greifswald, Germany; 3 Bruker BioSpin, Rheinstetten, Germany; 4 Section of Metabolic Genetics, Novo Nordisk Foundation Center for Basic Metabolic Research, University of Copenhagen, Copenhagen, Denmark; 5 Private Practice Endocrinology, Erfurt, Germany; 6 Department of Clinical Experimental Research, Rigshospitalet, Copenhagen, Denmark; 7 Department of Clinical Medicine, Faculty of Health and Medical Sciences, University of Copenhagen, Copenhagen, Denmark; National Research Council of Italy, ITALY

## Abstract

**Background:**

Triiodothyronine (T3) and thyroxine (T4) as the main secretion products of the thyroid affect nearly every human tissue and are involved in a broad range of processes ranging from energy expenditure and lipid metabolism to glucose homeostasis. Metabolomics studies outside the focus of clinical manifest thyroid diseases are rare. The aim of the present investigation was to analyze the cross-sectional and longitudinal associations of urinary metabolites with serum free T4 (FT4) and thyroid-stimulating hormone (TSH).

**Methods:**

Urine Metabolites of participants of the population-based studies Inter99 (n = 5620) and Health2006/Health2008 (n = 3788) were analyzed by ^1^H-NMR spectroscopy. Linear or mixed linear models were used to detect associations between urine metabolites and thyroid function.

**Results:**

Cross-sectional analyses revealed positive relations of alanine, trigonelline and lactic acid with FT4 and negative relations of dimethylamine, glucose, glycine and lactic acid with log(TSH). In longitudinal analyses, lower levels of alanine, dimethylamine, glycine, lactic acid and N,N-dimethylglycine were linked to a higher decline in FT4 levels over time, whereas higher trigonelline levels were related to a higher FT4 decline. Moreover, the risk of hypothyroidism was higher in subjects with high baseline trigonelline or low lactic acid, alanine or glycine values.

**Conclusion:**

The detected associations mainly emphasize the important role of thyroid hormones in glucose homeostasis. In addition, the predictive character of these metabolites might argue for a potential feedback of the metabolic state on thyroid function. Besides known metabolic consequences of TH, the link to the urine excretion of trigonelline, a marker of coffee consumption, represents a novel finding of this study and given the ubiquitous consumption of coffee requires further research.

## Introduction

A normal thyroid function is crucial to maintain physiological actions including growth, development as well as regulation of metabolism and thermogenesis [1, 2]. The thyroid hormones (TH), triiodothyronine (T3) and thyroxine (T4), are central components of the hypothalamic-pituitary-thyroid axis and are involved in a broad range of processes ranging from energy expenditure and lipid metabolism to glucose homeostasis [1]. The physiological importance of both THs is widely accepted as THs nearly affect every human tissue [2]. The unbound fraction of T4 (FT4)—only 0.03% of T4 is free in the circulation [2]—plays a key role in clinical decision making with regard to the thyroid function together with thyroid-stimulating hormone (TSH) [3, 4].

Mass spectrometry (MS) and nuclear magnetic resonance (NMR) spectroscopy based metabolomics are used to detect physiological or pathological changes in cells, tissues or body fluids and activity of metabolic pathways. In recent years, these techniques have been used to gain deeper insights in both thyroid cancer progression [5] and THs-mediated regulation of metabolism [6–11]. Experimental rodent studies have investigated the metabolic profile of hypothyroidism. The studies revealed that several urine and serum metabolites involved in energy metabolism, amino acid (AA) metabolism, sphingolipid metabolism and purine metabolism were altered after the induction of hypothyroidism [6, 7]. Furthermore, a decline in the metabolic activity in cerebellum in a hypothyroid state was reported [8]. Large scaled human studies are however rare [9–11]. An analysis based on targeted serum metabolomics of nearly 1500 euthyroid subjects found associations between FT4 levels, but not TSH, and acylcarnitines or phosphatidylcholines and concluded that FT4 is linked to an increased β-oxidation [9]. Another study using urine samples of over 3300 subjects further highlighted the distinct actions of FT4 and TSH on amino acid, methane or phospholipid metabolism [10]. Whereas FT4 showed associations with gluconeogenic AAs and metabolites of the methane metabolism which argues for a relevant role in energy metabolism, TSH was linked to urinary levels of tyrosine and hippurate suggesting a role in the phenylalanine and tyrosine metabolism. Furthermore, based on the same analytical approach, associations of urinary metabolites including trigonelline, pyroglutamate, acetone and hippurate with the TH metabolite 3,5-diiodo-L-thyronine (3,5-T2) were found as well [11].

The aim of the present investigation was to analyze the associations between 17 urinary metabolites and serum FT4 and TSH by using non-targeted ^1^H-NMR measurements in two population-based studies comprising over 9400 subjects.

## Material and methods

### Study populations

All population-based studies were performed at the Research Centre for Prevention and Health, The Capital Region of Denmark. Written informed consent was obtained from all participants and the Inter99 (KA 98155), Health2006 (KA-20060011) and Health2008 (H-KA20060011) were approved by the regional Ethical Committee and the Danish Data Protection Agency. Furthermore, the present project was approved by the Ethics Committee of the Capital Region of Denmark (H-15004167) and the Danish Data Protection Agency.

#### The Inter99 study

The Inter99 is a randomized population-based intervention study initiated in March 1999 and ended in April 2006. The study design and the intervention have been described in detail elsewhere [12] and can be found on the website http://www.inter99.dk. The individuals were drawn from the Civil Registration System. The study population (N = 61,301) comprised all individuals in specific age-groups (30, 35, 40, 45, 50, 55 and 60 years) from a defined area of Copenhagen. From this study population 13,016 persons were drawn for the intervention and 6784 participants (52.5%) accepted and were examined at baseline.

**Fig 1:**
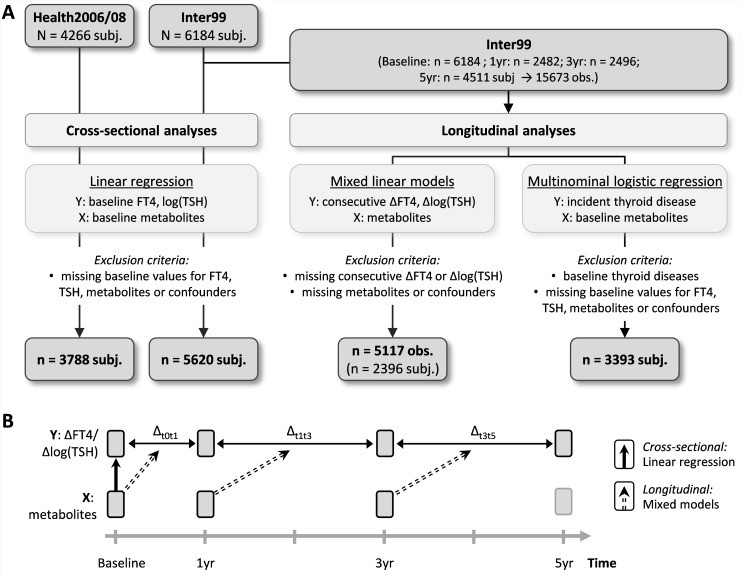
Study design. A) Flow diagram of analyses strategies. B) Graphical chart of cross-sectional and longitudinal analyses.

NMR measurements were available for 6184 of the baseline participants. For cross-sectional analyses, 289 subjects without FT4 and TSH measurements and additionally 258 subjects without information about confounding factors were excluded resulting in a final Inter99 study population of 5620 subjects. For longitudinal analyses two different statistical analyses were applied (Fig 1) leading to two different study populations after exclusion of missing data for FT4, TSH, metabolites or confounders: 1) association between metabolite levels and changes in FT4 and TSH (mixed linear regression: n = 5117 observations of 2396 subjects, using of baseline, 1-yr, 3-yr and 5-yr information) and 2) association between baseline metabolite levels and risk of hypo- and hyperthyroidism (multinomial logistic regression: n = 3393 subjects, using only subjects with complete baseline and 5-yr follow-up information and without baseline thyroid diseases).

#### The Health2006/08 study

Health2006 participants (N = 3471, participation rate: 44.7%) or Health2008 participants (N = 795) were recruited from June 2006 to May 2008 or September 2008 to December 2009 from the Danish Central Personal Register, as a random sample from persons living in the western area of the Capital Region of Denmark, respectively. Based on both studies, for 4000 participants NMR measurements were available. Of these subjects, 173 subjects without FT4 and TSH measurements and additionally 39 subjects without information about confounding factors were excluded. The final Health2006/08 study population comprised 3788 subjects for cross-sectional analyses.

### Measurements

#### The Inter99 study

Information on coffee consumption was assessed by questionnaire. Height and weight were measured without shoes and with light clothes. Body mass index (BMI) was calculated as weight (kg) divided by height (m) squared. All participants had their blood pressure measured twice with a mercury sphygmomanometer (Mercuro 300; Speidel & Keller GmbH & Co, Jungingen, Germany) with appropriate cuff size, after 5 min of rest, in the lying position, and the average of the recorded measurements was used. Hemoglobin A_1c_ (HbA_1c_) was measured for all participants by principles of ion exchange HPLC using Bio-Rad VARIANT ^™^ Hemoglobin A_1C_ (BioRad, USA). Low-density lipoprotein (LDL) cholesterol was measured using enzymatic colorimetric methods (Roche, Mannheim, Germany).

#### The Health2006/08 study

Height and weight were measured without shoes and with light clothes. Body mass index (BMI) was calculated as weight (kg) divided by height (m) squared. Blood pressure was measured in the sitting position after 5 minutes of rest using a Mercury300 mercury phygmomanometer, and the mean of two values was used for systolic and for diastolic pressure. Systolic blood pressure was read at the first Korotkoff sound and diastolic blood pressure at the disappearance of the Korotkoff sounds (phase V). The deflation rate was 2 mmHg/second. Before the investigation, the manometer was calibrated. Fasting blood samples were analyzed for HbA1c (BioRad, USA). LDL cholesterol was measured using enzymatic colorimetric methods (Roche, Mannheim, Germany).

### Thyroid hormones

For Inter99 and Health2006/08 serum TSH and FT4 concentrations were analyzed in the central laboratory of the University Medicine Greifswald by a homogeneous, sequential, chemiluminescent immunoassay based on LOCI^®^ technology (Dimension Vista^®^ System Flex^®^ reagent cartridge; Siemens Healthcare Diagnostics, Inc., Newark, DE). The analytical measuring range was 0.005–100 mIU/mL and 0.1–8.0 ng/dL for TSH and FT4, respectively. In Inter99, the coefficients of variance (CV) at low, medium and high levels were 2.77%, 2.46% and 2.28% as well as 3.15%, 2.84% and 3.32% for TSH, respectively. The CVs for FT4 at low, medium and high levels were 2.33%, 3.31% and 2.96% as well as 3.53%, 2.13% and 3.17% in Health2006 and Health2008, respectively. The CVs for TSH at low, medium and high levels were 3.71%, 4.10% and 5.15% as well as 2.96%, 3.89% and 4.58% in Health2006 and Health2008, respectively.

### ^1^H-NMR spectroscopic analysis of urinary specimens

After thawing, urine specimens were five minutes centrifuged at 3000g and the supernatant was used for spectroscopic analysis. Therefore, we mixed 450 μl urine with 50 μl phosphate buffer to stabilize urinary pH at 7.0 (±0.35). The buffer was prepared with D2O and contained sodium 3-trimethylsilyl-(2,2,3,3-D4)-1-propionate (TSP) as reference. Spectra were recorded at the University Medicine Greifswald, Germany, on a Bruker DRX-400 NMR spectrometer (Bruker BioSpin GmbH, Rheinstetten, Germany) operating at 1H frequency of 400.13 MHz and equipped with a 4-mm selective inverse flow probe (FISEI, 120 μl active volume) with z-gradient. Specimens were automatically delivered to the spectrometer via flow injection. The acquisition temperature was calibrated to 300 ± 0.1K. A standard one-dimensional ^1^H-NMR pulse sequence with suppression of the water peak (NOESYGPPR1D) was used: RD—P(90°)—4 μsec—P(90°)—tm—P(90°)—acquisition of the free induction decay (FID). For each sample, the non-selective 90° hard pulse P(90°) was individually calibrated in full automation using the Bruker automation program PULSECAL. The relaxation delay (RD), the mixing time (tm), and the acquisition time were set to 4 sec, 10 msec, and 3.96 sec, respectively, resulting in a total recycle time of ~8.0 sec. Low-power continuous-wave irradiation on the water resonance at an rf-field strength of 25 Hz was applied during RD and tm for pre-saturation. 1ms Z-Gradients were applied between RD and P(90°) and between tm and P(90°) to further reduce the residual solvent signal. After application of 4 dummy scans, NS 32 were collected into 65536 (64K) complex data points using a spectral width of 20.689 parts per million (ppm) and a receiver gain (RG) setting of 128. FIDs were multiplied with an exponential function corresponding to a line broadening of 0.3 Hz before Fourier-transformation. TopSpin Version TS2.1pl6 was generally used for automated data acquisition and data processing. Spectra were automatically phase corrected and automatically referenced to the internal standard (TSP– 0.0 ppm) using Bruker’s processing program APK0.NOE. 18 metabolites were automatically annotated and quantified using Bruker’s Remote Data Analysis Server. The quantification approach described in brief: Quantification is done via signal fitting using a simplex algorithm under consideration of constraints. Constraints are metabolite and signal specific parameters and their ranges, including: (1) metabolite parameters (molecular mass), (2) parameters/strategies for identification of each considered signal of each metabolite (number of protons, relaxation time, multiplicity, coupling constant and/or peak patterns, search range for signal detection), (3) definition of additional signal fit parameters, their start, and allowed minimum and maximum values (fit range, chemical shift, line width, coupling constant, Gauss-Lorentz-ratio, baseline offset and slope). Seventeen metabolites were used in the present study. To account for urine dilution, metabolite concentrations were normalized by urine creatinine levels (metabolites; reported as millimoles per mole of creatinine).

### Bucketing of ^1^H-NMR spectra

Processed spectra were segmented into n = 500 consecutive integrated spectral regions (buckets) of fixed bucket width (0.018 ppm), covering the range from 0.5 ppm to 9.5 ppm (R, R Foundation for statistical computing, version 3.0.1, Vienna, Austria). The 4.5–5.0 ppm chemical shift region (28 buckets) was left out of the analysis in order to remove effects of variations in the suppression of water resonance, and variations in the urea signal caused by partial cross-solvent saturation through solvent-exchanging protons. To account for urine dilution, buckets were normalized by urine creatinine levels.

### Statistical analysis

Continuous data are expressed as median (1^st^; 3^rd^ quartile); nominal data as percentage. For bivariate statistics the Wilcoxon-rank-sum test (continuous data) or χ2-test (nominal data) were used to compare men and women. For regression analyses, metabolites and buckets were log2-transformed. TSH concentrations were log-transformed to achieve a more normal-like distribution. The analysis strategy is displayed in Fig 1. In a first step, linear regression models were used to assess the cross-sectional associations between metabolites levels or buckets and FT4 or TSH concentrations in Inter99 and Health2006/08 cohorts. Inter99 was used as discovery cohort and results were dedicated significant if they could be replicated in Health2006/08. In a second step, two types of longitudinal analyses were performed based on the repeated measurements in Inter99: I) mixed effect linear models with random intercept were used to examine the longitudinal association between metabolites or buckets and changes in FT4 or log(TSH) over time (based on 2396 subjects with 5117 observations). II) Multinomial logistic regression models were calculated to assess the relation between baseline metabolite levels and thyroid diseases. Therefore, participants were categorized into three groups according to TSH levels: 1) sub/overt hypothyroidism (men: TSH > 3.3 mU/l; women: TSH > 4.9 mU/l), 2) euthyroid (reference group) or 3) sub/overt hyperthyroidism (TSH < 0.4pmol/l). Odds ratios with 95% confidence intervals (CI) were calculated. All models were adjusted for age, sex, BMI, HbA1c, LDL cholesterol and systolic blood pressure as well as additionally for coffee consumption in a second model (only Inter99 and cross-sectional analyses). In sensitivity analyses for the longitudinal mixed linear models we excluded all observation where changes in FT4 or TSH did not exceed a critical value known as the Reference Change Value (RCV) and thus were not significant. Variations in a laboratory analyt could be due to biological and analytical variation. The RCV is given as: $RCV=2^{½}*Z*{\left(CV_{A}^{2}+CV_{I}^{2}\right)}^{½}$ with CV_A_ = analytical impression, CV_I_ = within-subject biological variation and Z = number of standard deviation appropriate to the desired probability (in the present study Z = 1.645 for 90% significance). Based on biological variation for FT4 of 5.7% and for TSH of 19.3% [13] updated every two years: https://www.westgard.com/biodatabase1.htm] the calculated RCVs were 14.4% and 45.2% for FT4 and TSH, respectively. The number of used observation reduced from 5117 to 689 for FT4 and 1140 for TSH. To account for multiple testing, we adjusted the p-values by controlling the false discovery rate (FDR) at 5% using the Benjamini-Hochberg procedure. Statistical analysis were performed using SAS version 9.4 (SAS statistical software, version 9.4, SAS Institute, Inc; NC, USA).

## Results

### General characteristics

General characteristics of the study populations (cross-sectional analyses) are displayed in Table 1. Participants of Inter99 were on average 5 years younger, more often current smoker, had a higher BMI and systolic blood pressure as well as a more unfavorable lipid profile than Health participants. With respect to thyroid hormones, Inter99 subjects had higher FT4 levels and lower TSH levels compared to the Health2006/08 studies.

**Table 1:** General characteristics of the study populations.

	Inter99	Health2006/08	P*
	(n = 5620)	(n = 3788)	
Men, %	49.1	44.3	<0.01
Age, years	45 (40; 50)	50 (40; 59)	
Smoking, %			<0.01
Never smoker	35.3	42.4	
Ex-smoker	25.6	32.7	
Current smoker	39.2	24.9	
BMI, kg/m^2^	25.6 (23.2; 28.6)	25.1 (22.6; 28.1)	<0.01
Diabetes, %	1.8	3.2	<0.01
Systolic BP, mmHg	130 (120; 140)	126 (116; 138)	<0.01
Diastolic BP, mmHg	80 (75; 90)	80 (74; 88)	<0.01
Total cholesterol, mmol/l	5.4 (4.8; 6.2)	5.0 (4.4; 5.7)	<0.01
LDL cholesterol, mmol/l	3.4 (2.8; 4.1)	3.2 (2.6; 3.8)	<0.01
HDL cholesterol, mmol/l	1.4 (1.1; 1.7)	1.5 (1.2; 1.8)	<0.01
eGFR,	94 (84; 106)	83 (73; 94)	<0.01
FT4, pmol/l	15.0 (13.9; 16.3)	12.5 (11.5; 13.6)	<0.01
TSH, mU/l	1.25 (0.88; 1.78)	1.49 (1.03; 2.18)	<0.01
Coffee consumption, %			-
0 cup/day	12.0	-	
1 cup/day	6.0	-	
2 cup/day	10.7	-	
3 cup/day	11.5	-	
4 cup/day	13.0	-	
5 cup/day	12.7	-	
≥ 6 cup/day	34.0	-	

Continuous data are expressed as median (1st; 3rd quartile); nominal data as percentage.

*Wilcoxon-rank-sum test (continuous data) or χ2-test (nominal data).

HDL = high-density lipoprotein; LDL = low-density lipoprotein; BP = blood pressure; BMI = body mass index; FT4 = free thyroxine; TSH = thyrotropin.

### Cross-sectional association between metabolites and thyroid function

**Fig 2:**
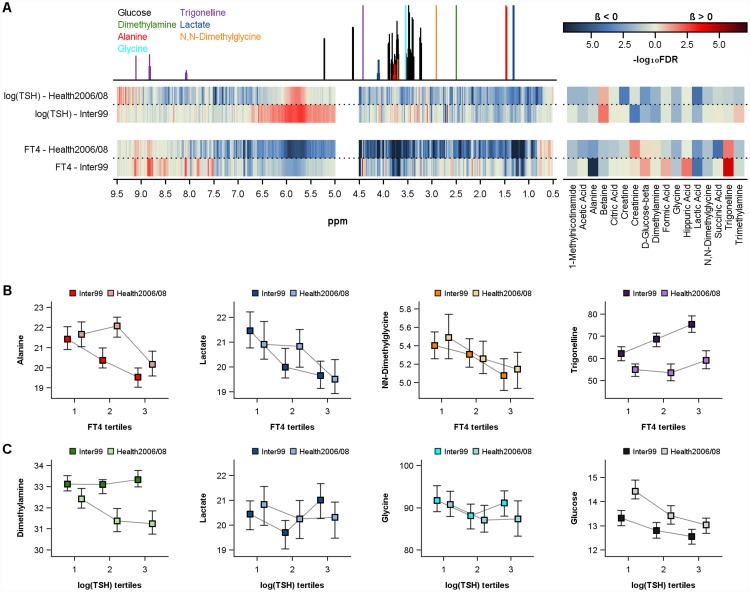
Cross-sectional association between urine metabolome and thyroid function. A) Corrected p-values (false discovery rate (FDR)) of the cross-sectional associations of metabolites levels (right side) or ppm (left side) with free thyroxine (FT4) or thyrotropin (TSH) in Inter99 and Health2006/08. Multivariable linear regression models were adjusted for age, sex, body-mass-index, HbA1c, low-density lipoprotein cholesterol and systolic blood pressure. B) Median levels of alanine, lactate, N,N-dimethylglycine or trigonelline by tertiles of FT4 in Inter99 and Health2006/08. C): Median levels of glycine, lactate, dimethylamine or glucose by tertiles of log(TSH) in Inter99 and Health2006/08.

Adjusted linear regression analyses revealed associations between several urine metabolites and serum FT4 levels with five metabolites including alanine, glycine, N,N-dimethylglycine, trigonelline and lactic acid were associated in both study populations (Table 2, Fig 2B). Furthermore, betaine was additionally associated in Health2006/08 and barely missed statistical significance in Inter 99 (FDR = 0.06). With the exception of trigonelline, all detected metabolites were negatively related to FT4. As trigonelline is known as a biomarker of coffee consumption we additionally adjusted the models in Inter99 for the reported daily coffee intake of the participants. However, the associations still remained significant [beta 0.102 (standard error 0.032); FDR<0.05]. With respect to ppm regions, the bucket-based analyses confirmed the above-mentioned metabolites by showing associations between metabolite-specific ppm regions and FT4 levels (Fig 2A). Furthermore, inverse relations with FT4 levels were found for several regions including 1.12–1.16, 1.22–1.25, 6.87–6.90 and 7.35–7.38 ppm in both study populations (S1 Fig). The doublet observed between 1.12 and 1.16 might be isobutyric acid [14] a metabolite derived from microbial activity. However, no such assignment was possible for the remaining spectral parts.

**Table 2:** Cross-sectional associations of urinary metabolites with FT4 or TSH concentrations in both study populations.

Metabolite	FT4				log(TSH)			
	Inter99		Health2006/08		Inter99		Health2006/08	
	Beta (SE)	FDR	Beta (SE)	FDR	Beta (SE)	FDR	Beta (SE)	FDR
Acetic Acid	-0.016 (0.028)	0.67	-0.074 (0.023)	0.01	-0.018 (0.008)	0.06	-0.024 (0.008)	0.02
Alanine	-0.267 (0.046)	<0.01	-0.147 (0.041)	<0.01	-0.021 (0.014)	0.23	-0.034 (0.015)	0.05
Betaine	-0.062 (0.027)	0.06	-0.061 (0.027)	0.04	0.023 (0.008)	0.01	0.018 (0.010)	0.11
Citric Acid	-0.007 (0.026)	0.87	-0.055 (0.027)	0.06	0.002 (0.008)	0.87	-0.006 (0.010)	0.62
Creatinine	0.029 (0.043)	0.67	0.095 (0.038)	0.03	-0.054 (0.013)	<0.01	-0.014 (0.014)	0.40
Creatine	0.000 (0.021)	0.98	-0.025 (0.016)	0.17	0.001 (0.006)	0.89	-0.026 (0.006)	<0.01
Dimethylamine	-0.048 (0.077)	0.67	0.034 (0.062)	0.62	-0.079 (0.023)	<0.01	-0.068 (0.023)	0.01
Formic Acid	0.068 (0.034)	0.10	-0.051 (0.034)	0.18	-0.009 (0.010)	0.56	-0.011 (0.013)	0.47
D-Glucose-beta	0.072 (0.033)	0.06	0.025 (0.035)	0.54	-0.028 (0.010)	0.01	-0.049 (0.013)	<0.01
Glycine	-0.062 (0.027)	0.05	-0.090 (0.030)	0.01	-0.023 (0.008)	0.01	-0.030 (0.011)	0.02
Hippuric Acid	0.087 (0.029)	0.01	-0.004 (0.027)	0.87	-0.008 (0.009)	0.56	-0.002 (0.010)	0.85
Lactic Acid	-0.183 (0.041)	<0.01	-0.140 (0.037)	<0.01	-0.035 (0.012)	0.01	-0.064 (0.014)	<0.01
N,N-Dimethylglycine	-0.098 (0.039)	0.04	-0.083 (0.035)	0.04	-0.007 (0.012)	0.67	-0.030 (0.013)	0.04
Succinic Acid	-0.024 (0.043)	0.67	-0.172 (0.036)	<0.01	0.005 (0.013)	0.80	-0.021 (0.014)	0.17
Trigonelline	0.112 (0.024)	<0.01	0.065 (0.023)	0.02	-0.006 (0.007)	0.57	-0.015 (0.009)	0.11
Trimethylamine	-0.047 (0.032)	0.25	-0.071 (0.031)	0.04	0.014 (0.010)	0.23	-0.025 (0.011)	0.05
1-Methylnicotinamide	-0.082 (0.045)	0.13	-0.105 (0.044)	0.04	-0.012 (0.014)	0.58	-0.046 (0.017)	0.02

FDR = false discovery rate. SE = standard error; FT4 = free thyroxine; TSH = thyroid-stimulating hormone. Linear regression models were adjusted for age, sex, body-mass-index, HbA1c, LDL cholesterol and systolic blood pressure.

With respect to TSH, four urine metabolites including dimethylamine (DMA), D-glucose-beta, glycine and lactic acid were significantly (FDR<0.05) and inversely associated in both study populations (Table 2, Fig 2C). Furthermore, creatinine and betaine, or acetic acid, creatine and 1-methylnicotinamide were related to TSH levels in Inter99 or Health2006/08, respectively. No clear associations to ppm regions based on bucket analyses become apparent in both populations.

### Longitudinal association between metabolites and thyroid function

**Fig 3:**
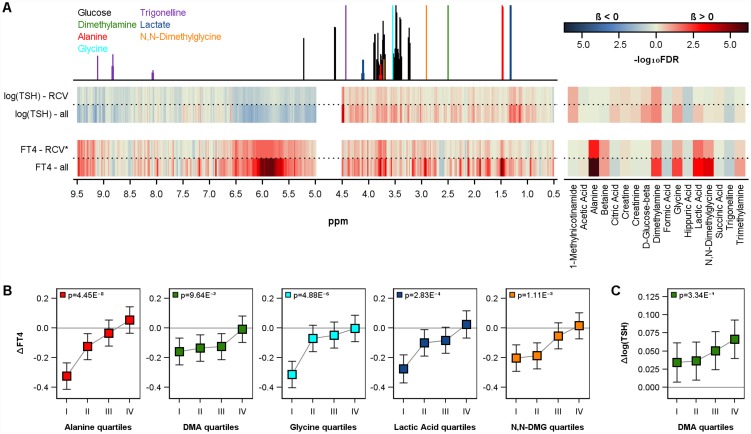
Longitudinal association between urine metabolome and thyroid function. A) Corrected p-values (false discovery rate (FDR)) of the longitudinal associations of urine metabolites levels (right side) or ppm (left side) with changes in free thyroxine (FT4) or thyrotropin (TSH) in Inter99. Models were performed in all subjects (x-axis) and *only in subjects with noticeable change assessed by the reference change value (RCV). Multivariable mixed linear models were adjusted for age, sex, body-mass-index, HbA1c, low-density lipoprotein cholesterol and systolic blood pressure (for more details see method section). Estimated mean value with 95% confidence limits for changes in free thyroxine (B: ΔFT4) or thyrotropin (C: Δlog(TSH)) by quartiles of selected urinary metabolite concentrations in Inter99 calculated by mixed linear models. DMA = dimethylamine. DMG = dimethylglycine.

The longitudinal associations between urine metabolites and thyroid function were tested by mixed linear regression models based on Inter99 data. In general, a decline in mean FT4 and an increase in log(TSH) levels were observed between the baseline [FT4 15.14 pmol/l); TSH 1.64 mU/l] and 5 year follow-up [FT4 14.84 pmol/l); TSH 1.86 mU/l] possibly indicating a worsening of the hypothalamic-pituitary-thyroid axis mediated feedback with increasing age. Urinary level of alanine, DMA, glycine, lactic acid and N,N-dimethylglycine were significantly (FDR<0.05) associated to changes in FT4 levels (Table 3, Fig 3A). In detail, for all mentioned metabolites lower levels were related to a higher decline in FT4 levels over time (Fig 3B). With respect to TSH levels, mixed model analyses revealed significant associations with DMA (Table 3, Fig 3C).

**Table 3:** Longitudinal associations of urinary metabolites with changes in FT4 or TSH concentrations in Inter99.

Metabolite	FT4				Log(TSH)			
	All subjects		subjects with noticeable change assessed by the RCV		All subjects		subjects with noticeable change assessed by the RCV	
	Beta (SE)	FDR	Beta (SE)	FDR	Beta (SE)	FDR	Beta (SE)	FDR
Acetic Acid	0.010 (0.019)	0.70	0.038 (0.103)	0.90	0.000 (0.006)	0.96	-0.017 (0.020)	0.62
Alanine	0.223 (0.034)	<0.01	0.792 (0.188)	<0.01	-0.001 (0.010)	0.93	-0.009 (0.038)	0.91
Betaine	0.045 (0.020)	0.09	0.291 (0.112)	0.09	-0.004 (0.006)	0.70	-0.005 (0.023)	0.91
Citric Acid	-0.032 (0.020)	0.21	-0.101 (0.116)	0.62	0.008 (0.006)	0.29	0.021 (0.022)	0.61
Creatinine	-0.020 (0.031)	0.70	-0.044 (0.184)	0.91	0.011 (0.009)	0.42	0.025 (0.035)	0.70
Creatine	0.007 (0.016)	0.73	0.080 (0.087)	0.61	0.004 (0.005)	0.54	0.020 (0.018)	0.48
Dimethylamine	0.201 (0.057)	<0.01	0.701 (0.293)	0.10	0.046 (0.017)	0.04	0.159 (0.058)	0.08
Formic Acid	-0.047 (0.025)	0.14	-0.215 (0.139)	0.38	-0.006 (0.007)	0.60	-0.034 (0.027)	0.45
D-Glucose-beta	-0.013 (0.025)	0.70	-0.014 (0.125)	0.97	0.017 (0.007)	0.09	0.034 (0.026)	0.45
Glycine	0.073 (0.022)	0.01	0.261 (0.122)	0.15	0.015 (0.006)	0.09	0.032 (0.026)	0.45
Hippuric Acid	-0.028 (0.021)	0.35	-0.157 (0.122)	0.45	-0.013 (0.006)	0.11	-0.051 (0.025)	0.15
Lactic Acid	0.121 (0.031)	<0.01	0.540 (0.164)	0.02	0.013 (0.009)	0.29	0.002 (0.032)	0.97
N,N-Dimethylglycine	0.128 (0.030)	<0.01	0.408 (0.162)	0.09	0.003 (0.009)	0.79	-0.020 (0.035)	0.76
Succinic Acid	-0.034 (0.032)	0.48	-0.070 (0.172)	0.90	0.004 (0.010)	0.73	0.023 (0.036)	0.74
Trigonelline	-0.037 (0.019)	0.13	-0.192 (0.106)	0.25	-0.010 (0.006)	0.16	-0.025 (0.020)	0.45
Trimethylamine	0.055 (0.025)	0.09	0.193 (0.139)	0.45	0.004 (0.007)	0.70	-0.009 (0.029)	0.91
1-Methylnicotinamide	-0.020 (0.037)	0.70	0.002 (0.200)	0.99	0.022 (0.011)	0.14	0.087 (0.041)	0.15

FDR = false discovery rate. SE = standard error; FT4 = free thyroxine; TSH = thyroid-stimulating hormone; RCV = reference change value (RCV; p = 0.1). Mixed linear models were adjusted for age, sex, body-mass-index, HbA1c, LDL cholesterol and systolic blood pressure.

Longitudinal analyses were further repeated after the exclusion of all observations which did not achieved the critical RCV of 14.4% for FT4 or 45.2% for TSH (see method section). The above-described associations between urinary levels of alanine, DMA, lactic acid or N,N-dimethylglycine and FT4 levels as well as between DMA and TSH were confirmed even if the FDR-values increased due to the substantially reduced number of observations (Table 3, Fig 3).

Multinomial logistic regression (S1 Table) showed that subjects with higher trigonelline [per SD increase: OR 1.38 (95%-CI 1.14–1.67), p<0.01, FDR = 0.03] or hippuric acid [per SD increase: OR 1.27 (95%-CI 1.08–1.50), p<0.04, FDR = 0.07] as well as lower lactic acid [per SD increase: OR 0.51 (95%-CI 0.31–0.81), p = 0.01, FDR = 0.07], alanine [per SD increase: OR 0.71 (95%-CI 0.51–0.91), p = 0.02, FDR = 0.18] or glycine [per SD increase: OR 0.61 (95%-CI 0.31–1.00), p = 0.04, FDR = 0.24] levels at baseline had an increased risk of hypothyroidism (n = 83). No significant association with hyperthyroidism was revealed mainly due to the low number of cases (n = 41).

## Discussion

The present study investigated metabolic alterations in relation to thyroid function. For this purpose, we analyzed the cross-sectional relation between urinary metabolite concentrations and FT4 or log(TSH) as well as the longitudinal association to changes in the thyroid function in two independent populations.

### Amino acid metabolism

Cross-sectional analyses revealed negative associations of FT4 levels with the urinary levels of the glucogenic AAs alanine and glycine as well as lactic acid, betaine and N,N-dimethylglycine in both study populations. Furthermore, urinary glycine, lactic acid and glucose levels were inversely related to log(TSH). Previous metabolomics studies already linked urinary excretion of glycine, alanine and lactic acid with thyroid function by reporting inverse associations with FT4 in cross-sectional analyses. In line with the cross-sectional positive association of FT4 (p = 0.03) as well as inverse association of log(TSH) (p<0.01) with urinary glucose in Inter99, former studies already showed a major role of THs in the regulation of glucose homeostasis by e.g. increasing gluconeogenesis in the kidney and liver but also glucose absorption and utilization in target cells [1, 15]. This was confirmed by higher glucose levels in patients with hyperthyroidism and a complex interplay between diabetes and thyroid disease. Furthermore, an experimental study in rats demonstrated that T4 administration leads besides an increased rate of glucose production in isolated livers, also leads to an increased transport of alanine in hepatocytes arguing for an enhanced conversion of alanine into glucose [16]. Studies in humans confirmed these findings by detecting lower blood levels of alanine and glycine in hyperthyroidism [17] or after T3 administration [18] in combination with an increased uptake of these amino acids. The higher uptake of glucogenic AAs might be one reason for the detected decreasing urinary excretion of alanine and glycine with increasing FT4. A further reason might be that the FT4 mediated elevated gluconeogenesis rate and thus consequently increased demand of gluconeogenic precursors resulted in an increased renal resorption of amino acids like alanine or glycine. THs were shown to have direct renal effects e.g. on glomerular filtration rate and re-absorptive processes. With respect to the latter point, THs influence the expression and/or activity of various renal channels and transporters [19]. However, not all studies confirmed decreased serum or urine glucogenic AAs in hyperthyroidism. Among 25 women with thyrotoxicosis the sum of serum glucogenic AAs was elevated, whereas no alteration was found in women with hypothyroidism compared to euthyroid controls [20]. Moreover, a population-based cohort even found no association between FT4 levels and serum levels of glucogenic AAs including glycine, valine, serine or proline in euthyroid subjects [9]. Interestingly in contrast to the cross-sectional findings in the present study, longitudinal analyses revealed that subjects with high baseline urinary levels of alanine, glycine and lactate exhibit a smaller decrease or even an increase in FT4 over time in Inter99. One explanation for these discrepancies might be the initial lower FT4 levels in subjects with high metabolite levels at baseline (cross-sectional analyses). However, the adjustment for baseline FT4 levels did not changed the results substantially (data not shown). Given the general worsening of the hypothalamic-pituitary-thyroid axis mediated feedback over time this observation may imply a change in this system based on the metabolic state of the individual, e.g. high urine levels of alanine predict an increase in FT4 whereas low levels predict a decrease over time. The change in FT4 could be seen as a response to the metabolic state of the individual rather than reflecting the actual effect of FT4 on metabolism. Following this suggestions, urine metabolome analyses provide potential to predict the individual thyroid state. Unfortunately, the low number of thyroid disease in the present study population limits the evaluation of the clinical utility of these findings.

Besides increased uptake of glycine for gluconeogenesis even a decreased production could account for this finding. In this respect the aligned change in betaine and N,N-dimethylglycine in cross-sectional and longitudinal analyses are of particular interest. Betaine-homocysteine S-methyltransferase facilitates the conversion of homocysteine to methionine requiring betaine as methyl donor and producing dimethylglycine, which could be further converted to sarcosine and finally to glycine *via* sarcosine dehydrogenase activity. Interestingly, a suppressive effect of TH in animal models on (hepatic) betaine-homocysteine S-methyltransferase activity was reported [21, 22]. However, the suspected increased availability of betaine might be blunted through a general down regulation of this pathway.

### Coffee metabolism

Beside metabolites linked to amino acid metabolism, a strong positive association between urinary trigonelline levels and FT4 was detected. An association with the urine excretion of trigonelline was previously reported with respect to the TH metabolite 3,5-T2 in a large sample of euthyroid subjects [11]. The formation of 3,5-T2 in vivo is still under debate but early studies suggested its endogenous origin from either T3 or T4, mainly as an inactivating step in the local control of T3 action [23]. It has to be noted, that up to now no clear relation between 3,5-T2 and the classical TH is established in humans [24]. Given the disappearance of this strong association in the longitudinal analyses an altering effect of trigonelline in the conversion or clearance of TH is likely. Trigonelline is known as an metabolite in the niacin metabolism and is found in many plants including fenugreek seeds, oat or potatoes and further represents a bioactive compound in coffee with a concentration ~1% [25]. Studies suggest that trigonelline is not substantially metabolized in the human body and is mostly excreted in urine [25, 26]. Therefore trigonelline is listed as an urinary marker of coffee consumption [27]. Regarding thyroid function it is suggested that coffee consumption altered the intestinal absorption of L-T4, the synthetic thyroid hormone equivalent used to treat hypothyroidism. Among several patients a lower incremental rise of serum T4 and a missing or incomplete normalization of TSH following oral L-T4 administration together with coffee were observed [28–30]. Unfortunately no further research regarding trigonelline and thyroid health is available up to now. Therefore, we could only speculate about the reason of the association between trigonelline and FT4. Coffee represents a complex mixture of more than 1000 partly bioactive compounds including beside caffeine and its metabolites paraxanthine, theobromine and theophylline, a high number of further molecules like trigonelline, phenolic acids, kahweol or cafestol [25]. Furthermore, urinary hippuric acid levels, as possible metabolite of coffee components, were revealed to be associated with coffee consumption [31] and showed the same association with FT4 levels as trigonelline in the Inter99 cohort. During the last decade several investigations dealt with the pleiotropic effects of coffee and/or its components on the metabolism and health status [32, 33]. Experimental studies suggested overlapping actions of caffeine/coffee [32–34] with THs [1, 15, 35] including increased metabolic rate, increased blood pressure or reduced insulin sensitivity. However, in general these caffeine/coffee specific effects are subject of a controversial debate and were not always confirmed. Regarding THs, an over 30 year old study investigated the effect of theophylline administration every 12 hour for 60 hour on the pituritary-thyroid axis in four healthy adults [36]. Plasma total T4 levels showed a simultaneous increase with theophylline levels over the whole experiment time and were accompanied by an increase in plasma as well as urinary cyclic adenosine 3',5'-monophosphate (cAMP) levels. The authors suggested that a theophylline mediated increase in intracellular cAMP led to the observed increase in T4 production. It has to be noted, that within the same study children treated with theophylline because of asthma showed a transient increase in plasma T4, which would fit to the disappearance of the FT4—trigonelline association in longitudinal analyses. Moreover, an expected hyperthyroid state leading to a hypermetabolic state was attenuated by an increased peripheral conversion of T4 to reverse T3, a less metabolic active TH metabolite. Implying even 3,5-T2 as metabolic degradation product of T4 this might link the previously mentioned association between 3,5-T2 and urinary trigonelline. However, theophylline is assumed to inhibit cyclic nucleotide phosphodiesterase and prevent the breakdown of cAMP which consequently lead to increased cAMP levels [37]. cAMP plays a major role in thyroid action. The binding of TSH to the G protein-coupled TSH receptor stimulates among others the cAMP production resulting in TH production and secretion via various intra-cellular mechanisms. Theophylline represents only one metabolite of caffeine in the group of methylxanthine and it remains unclear if other metabolites exhibit similar actions.

### Dimethylamine (DMA)

In the present study, urinary DMA was inversely associated with log(TSH) in cross-sectional analyses. Furthermore, low urinary DMA levels were related to a higher increase in log(TSH) and a higher decrease in FT4 over time. DMA is one of the most abundant components of the human urine and exogenous as well as endogenous sources of DMA are known [38]. Food components including trimethylamine, trimethylamine N-oxide, lecithin and choline have been found to increase urinary excretion of DMA and in particular after the consumption of fish and seafood an urinary DMA rise was observed [38, 39]. These metabolites are degraded by microbes yielding DMA. Interestingly, even the putative isobutyric acid (doublet between 1.12 and 1.16 ppm) is a proxy of microbial activity as fermentation product of dietary fibers. An influence of the thyroid hormone state on the microbiome might be mediated by altered bile acid production [40] which are well known to affect the composition of the gut microbiome [41]. Moreover, endogenous asymmetric dimethylarginine (ADMA), known as inhibitor of nitric oxide (NO) synthesis, can be directly degrade by dimethylaminohydrolase (DDAH) to the renally cleared products DMA and citrulline [42–44]. It is suggested that ADMA represents one major source for urinary DMA [45]. A recently published population-based study revealed that ADMA was cross-sectional related to low TSH levels as well as positively related with FT4 [46]. These results were confirmed in patients with Graves' disease [47] or hyperthyroidism [48, 49] and therefore fit in with our longitudinal findings of a higher decrease in FT4 levels among subjects with low DMA. Furthermore, experimental studies confirmed a relationship between ADMA and thyroid function by showing a down-regulation of the *DDAH1* gene in mice liver following T3 treatment [50] as well as the production of endogenous ADMA in primary human thyrocytes [51]. Beside ADMA the consumption of fish and seafood represent one source of DMA and therefore DMA might be marker of the dietary intake of iodine. A large Danish study could show that fish account for around 15% of iodine intake in adults [52] and it is well accepted that iodine is an essential element to thyroid function and in particular the production of thyroid hormone [53]. Thus one can speculate that a higher DMA levels are related to higher fish consumption and consequently to higher iodine intake and a better thyroid function. However these assumptions are speculative and further research is needed to identify behind the association of high DMA with a lower decrease of FT4 over time.

## Conclusion

The present study demonstrated associations of thyroid function with amino acids including glycine, betaine or N,N-dimethylglycine as well as glucose or lactic acid, and therefore supports previous studies arguing for an important role of THs in the regulation of glucose homeostasis. In addition, the predictive character of these metabolites might argue for a potential feedback of the metabolic state on thyroid function. Furthermore, the urine excretion of trigonelline, a marker of coffee consumption, was linked to the thyroid function. However, the coffee specific effects on thyroid health are unknown up to now and need to be further investigated keeping in mind the ubiquitous consumption of the beverage.

## Supporting information

S1 Table Associations of urinary baseline metabolites with risk of hypothyroidism and hyperthyroidism in Inter99. (PDF)

S1 Fig NMR spectra by Free Thyroxine (FT4) levels. Black spectrum: exemplary urinary median nuclear magnetic resonance spectrum based on 5000 buckets. Median spectrum for selected ppm regions by FT4 groups in Inter99 (above the exemplary spectrum) and Health2006/08 (below the exemplary spectrum).
(PDF)
