# Supplementary material for: Urinary metabolomics reveals glycemic and coffee associated signatures of thyroid function in two population-based cohorts
Source: PLoS One. 2017 Mar 2;12(3):e0173078. doi: 10.1371/journal.pone.0173078 (PMC5333857; doi:10.1371/journal.pone.0173078)
Supplement: S1 Table — (PDF) [file pone.0173078.s001.pdf]

S1 Table. Associations of urinary baseline metabolites with risk of hypothyroidism and hyperthyroidism in Inter99.

Metabolite	Hypothyroidism			Hyperthyroidism		
	OR (95%-CI) per SD increase	P	FDR	OR (95%-CI) per SD increase	P	FDR
Acetic Acid	0.91 (0.71; 1.21)	0.56	0.94	1.01 (0.81; 1.31)	0.82	0.94
Alanine	0.71 (0.51; 0.91)	0.02	0.18	1.21 (0.91; 1.51)	0.24	0.83
Betaine	1.01 (0.91; 1.31)	0.65	0.94	0.61 (0.21; 1.81)	0.37	0.94
Citric Acid	1.11 (0.81; 1.31)	0.69	0.94	1.01 (0.71; 1.41)	0.86	0.94
Creatinine	1.01 (0.81; 1.31)	0.94	0.94	1.11 (0.81; 1.51)	0.68	0.94
Creatine	0.91 (0.71; 1.21)	0.44	0.94	1.01 (0.81; 1.41)	0.89	0.94
Dimethylamine	1.01 (0.91; 1.31)	0.68	0.94	1.01 (0.71; 1.41)	0.92	0.94
Formic Acid	1.11 (0.91; 1.41)	0.32	0.90	1.01 (0.81; 1.41)	0.79	0.94
D-Glucose-beta	0.91 (0.51; 1.71)	0.74	0.94	0.91 (0.51; 1.61)	0.73	0.94
Glycine	0.61 (0.31; 1.00)	0.03	0.24	1.11 (0.91; 1.31)	0.49	0.94
Hippuric Acid	1.31 (1.11; 1.51)	<0.01	0.07	0.81 (0.51; 1.11)	0.18	0.70
Lactic Acid	0.51 (0.31; 0.81)	0.01	0.07	1.11 (1.01; 1.31)	0.15	0.69
N,N-Dimethylglycine	0.81 (0.61; 1.11)	0.16	0.69	0.81 (0.61; 1.21)	0.27	0.85
Succinic Acid	1.11 (0.91; 1.41)	0.39	0.94	1.01 (0.71; 1.41)	0.92	0.94
Trigonelline	1.41 (1.11; 1.71)	<0.01	0.03	0.71 (0.51; 1.11)	0.10	0.55
Trimethylamine	0.91 (0.71; 1.21)	0.42	0.94	1.01 (0.71; 1.31)	0.80	0.94
1-Methylnicotinamide	1.01 (0.91; 1.21)	0.64	0.94	1.01 (0.51; 1.91)	0.90	0.94

FDR = false discovery rate. OR = odds ratio; CI = confidence interval; SD = standard deviation. Multinominal logistic regression model (reference group = euthyroid) were adjusted for age, sex, body-mass-index, HbA1c, LDL cholesterol and systolic blood pressure.
