# Supplementary material for: Urinary metabolomics reveals glycemic and coffee associated signatures of thyroid function in two population-based cohorts
Source: PLoS One. 2017 Mar 2;12(3):e0173078. doi: 10.1371/journal.pone.0173078 (PMC5333857; doi:10.1371/journal.pone.0173078)
Supplement: S1 Fig — Black spectrum: exemplary urinary median nuclear magnetic resonance spectrum based on 5000 buckets. Median spectrum for selected ppm regions by FT4 groups in Inter99 (above the exemplary spectrum) and Health2006/08 (below the exemplary spectrum). (PDF) [file pone.0173078.s002.pdf]

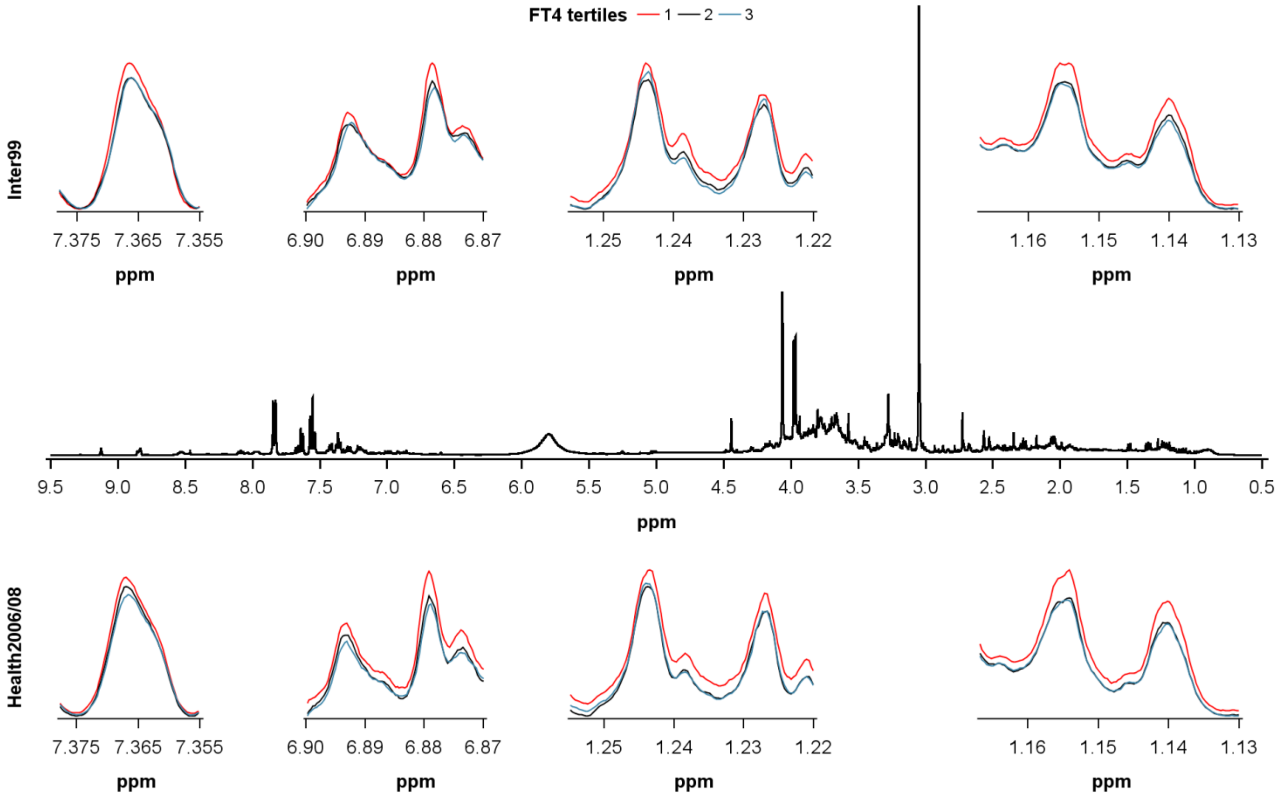

S1 Figure. NMR spectra by free thyroxine (FT4) levels. Black spectrum: exemplary urinary median nuclear magnetic resonance spectrum based on 5000 buckets. Median spectrum for selected ppm regions by FT4 groups in Inter99 (above the exemplary spectrum) and Health2006/08 (below the exemplary spectrum).
